# Neighborhood Greenspace and Cognition: The Cardiovascular Health Study

**DOI:** 10.1093/geroni/igab046.1182

**Published:** 2021-12-17

**Authors:** Sara Godina, Andrea Rosso, Gina Lovasi, Lilah Besser, Jana Hirsch, Jonathan Platt, Annette Fitzpatrick, Yvonne Michael

**Affiliations:** 1 University of Pittsburgh, Pittsburgh, Pennsylvania, United States; 2 Drexel Dornsife School of Public Health, Philadelphia, Pennsylvania, United States; 3 Florida Atlantic University, Boca Raton, Florida, United States; 4 Drexel University, Philadelphia, Pennsylvania, United States; 5 Columbia University Mailman School of Public Health, New York, New York, United States; 6 University of Washington, Seattle, Washington, United States; 7 Drexel University, PHILADELPHIA, Pennsylvania, United States

## Abstract

Access to greenspace has been positively associated with cognition among older adults, however prior research has been limited in temporal and geographic scope. We evaluated associations between neighborhood greenspace and incidence of dementia and change in cognitive functioning using a longitudinal sample of non-demented adults (n=2,465) from the Cardiovascular Health and Cognition Study. Percent greenness (1-km radial buffers) was derived from the National Land Cover Dataset. Cognitive function was measured using the Mini-Mental State Examination (3MSE) and dementia status was clinically adjudicated. Cox proportional hazard and logistics regression analyses were used to examine associations of baseline greenness with risk of incident dementia and risk of mild cognitive impairment, respectively. Generalized linear mixed models accounting for within-subject correlations were used to examine the association between greenspace in the neighborhood at baseline and 3MSE score (1991-1999). Ongoing results will be presented, along with modifiers and mediators of associations.

